# Enhanced Osteogenesis by Combining Exogenous BMPs with Hydroxyapatite/Aragonite Bone Grafts: In Vitro and In Vivo Studies

**DOI:** 10.3390/jfb16100361

**Published:** 2025-09-26

**Authors:** Hong Lu, Ines Sousa dos Santos, Emma Steijvers, Miriam Lazim, Victoria Higginbotham, Baichuan Wang, Zengwu Shao, Venkateswarlu Kanamarlapudi, Zhidao Xia

**Affiliations:** 1 Swansea University Medical School, Faculty of Medicine, Health and Life Science, Swansea University, Swansea SA2 8PP, UK; 849578@swansea.ac.uk (H.L.); 850700@swansea.ac.uk (I.S.d.S.); miriam.jones@swansea.ac.uk (M.L.); v.higginbotham@swansea.ac.uk (V.H.); k.venkateswarlu@swansea.ac.uk (V.K.); 2 Department of Physiology, Development and Neuroscience, University of Cambridge, Cambridge CB2 3EG, UK; es2025@cam.ac.uk; 3 Union Hospital, Tongji Medical College, Huazhong University of Science and Technology, Wuhan 430022, China; 2012xh0929@hust.edu.cn (B.W.); 1985xh0536@hust.edu.cn (Z.S.)

**Keywords:** osteogenesis, biomaterials, bone morphogenic proteins, BMP2, BMP7, mesenchymal stem cells, HEK293T cells, hydroxyapatite/aragonite, HAA

## Abstract

Synthetic biomaterials are widely used as bone graft substitutes, but their osteogenic capacity is limited as they lack osteogenic growth factors. This study aimed to enhance the osteogenesis of a novel hydroxyapatite/aragonite (HAA) biomaterial by incorporating decellularized bone matrix and bone morphogenetic protein (BMP)-2 and BMP-7 (BMP-2/7). Human umbilical mesenchymal stem cells (MSCs) were able to proliferate and differentiate on HAA. HEK-293T cells exogenously expressing BMP-2/7 successfully secreted BMP-2/7, which was assessed by enzyme-linked immunosorbent assay. By establishing a co-culture of MSCs with HEK-293T cells expressing BMP-2/7, we successfully created an artificial allograft that integrates both synthetic biomaterials and functional organic components, offering the potential to enhance osteogenesis. The decellularized (by freeze/thawing) functional HAA was implanted between the tibia and anterior tibialis muscle in murine models and assessed the induced bone formation via micro-computer tomography, histology, and osteogenic markers mRNA expression by a reverse transcription-quantitative polymerase chain reaction. A significant increase in new bone formation was seen in the functional HAA implanted group. In conclusion, this study revealed that bone formation following the HAA implantation can be enhanced by a functional decellularized matrix, comprising BMP-2/7, via in vitro tissue engineering using MSCs and HEK-293T cells expressing BMP-2/7.

## 1. Introduction

Bone grafts play a significant role in the restoration of extensive bone defects resulting from trauma, disease, or surgical interventions. However, the conventional sources of bone grafts, such as autografts and allografts, have limitations, including donor site morbidity and the risk of immunological rejection [1]. This has spurred the exploration of alternative solutions that can overcome these challenges while promoting effective bone regeneration [2,3,4]. In this context, the integration of synthetic scaffolds, particularly those composed of hydroxyapatite (HA) and calcium carbonate (CaCO_3_), has garnered attention for their biocompatibility and bioresorbable nature. Aragonite, which is one of the naturally occurring crystal forms of CaCO_3_, has very good biocompatibility but is not suitable for bone graft on its own due to its fast biodegradation ability [5].

HA, a key component of natural bone, provides a biomimetic environment that facilitates cell adhesion, proliferation, and differentiation [6,7,8]. However, conventionally used synthetic HAs are primarily ceramics with crystalline structures that differ from that of natural bone, and they tend to be slowly biodegradable. The addition of CaCO_3_ to HA can improve biodegradation and bone formation [9]. However, in comparison with allografts, synthetic bone biomaterials lack organic components that may enhance tissue integration and induce new bone formation. For instance, their osteogenic capacity is limited as they lack osteogenic growth factors [10]. Therefore, the inclusion of osteogenic growth factors such as bone morphogenic proteins (BMPs) in synthetic bone biomaterials can address this concern.

BMP-2 is known for its ability to stimulate osteoblastic differentiation and enhance bone regeneration, while BMP-7 exhibits multifaceted functions, including promoting osteogenic differentiation and inhibiting adipogenesis [11,12,13]. The incorporation of BMPs into synthetic bone biomaterials can induce osteogenic differentiation of mesenchymal stem cells (MSCs) and promote bone matrix formation. Harnessing the potential of BMPs within the context of tissue engineering offers a unique opportunity to accelerate and enhance the osteogenic capabilities of synthetic scaffolds. There are various approaches to deliver BMPs to induce bone formation. The first approach involves using a demineralized bone matrix (DBM) from animal or human sources. DBM may contain approximately 22 ng BMP-2, 6 ng BMP-4, and 85 ng BMP-7 per gram of human bone [14,15]. The second approach is to directly deliver recombinant BMPs to bone. However, the dose needed to reach clinical effects is extremely high, 4–40 mg per case [16]. Further, high-dose BMPs may cause ectopic bone formation and other risks. Another approach is to use carriers for the controlled release of BMPs, which require sophisticated technologies [17]. Lastly, MSCs from the host or a donor transformed with BMPs are a potential method for delivering BMPs to induce bone formation. However, human MSCs have proven difficult to transform to express BMPs [18].

It has been hypothesized in this study that artificial allografts can be developed by engineering synthetic biomaterials to contain decellularized tissue made from human cells transformed to have in vitro sources of BMPs. This was accomplished by establishing a co-culture of novel HA/aragonite (HAA) scaffolds [5,19], composite bone grafts created by incorporating aragonite into porous HA to enhance its biodegradation while maintaining optimal osteogenic capacity, along with MSCs and the Human Embryonic Kidney (HEK)-293T cells exogenously expressing BMP-2 and BMP-7 (HAA-MSC-HEK particles). The transfection of HEK-293T cells with BMP-2 and BMP-7 expression plasmids (BMP-2/7) resulted in the secretion of these proteins at high concentrations, which was confirmed by ELISA. HEK-293T cells expressing BMP-2/7 were co-cultured with MSCs on HAA scaffolds. After 3 days, the attachment cells to the scaffolds were assessed using ELISA, a real-time quantitative polymerase chain reaction (RT-qPCR), and confocal microscopy. In the second part of the study, HAA-MSC-HEK scaffolds were decellularized, sterilized, and implanted into the tibia of mice. This is to evaluate in vivo the time-dependent bone formation by micro-computed tomography (Micro-CT) imaging, histological staining of non-decalcified tissue sections, and RT-qPCR analysis of osteogenesis-related gene expression.

## 2. Materials and Methods

### 2.1. Human Umbilical Cord Mesenchymal Stem Cell (MSC) Isolation

Human umbilical cord tissue was obtained from Singleton Hospital (Swansea, UK) with the full informed consent of anonymized donors (West Wales Research Ethics Committee REC11/WA/0040). After removing the blood vessels, the umbilical cord tissues were washed with DMEM/F12 serum and antibiotic-free cell culture medium (ThermoFisher, Waltham, MA, USA) (catalogue number [CAT#] 11320033) to remove the blood and then sliced into 1–2 mm fragments. These tissue fragments were placed into a T25 culture flask and incubated in a humid incubator at 37 °C with 5% CO_2_ for 5 min to facilitate tissue attachment. Following this, 2 mL of foetal bovine serum (FBS [Sigma-Aldrich, St. Louis, MO, USA, CAT#F7524]) was added to the adhered tissue in the flask. After 24 h of incubation at 37 °C/5% CO_2_, the FBS was replaced with 3 mL of the full serum medium (DMEM/F12 supplemented with 10% FBS and 1% penicillin/streptomycin [PS], CAT#P4333). Flasks were monitored regularly for the emergence of new MSC outgrowths, and the full serum medium was replaced every 3–4 days until cells were ready for subculture or use.

### 2.2. HAA Particle Preparation

An HAA mix was prepared by combining a 1:1 molar ratio of tetracalcium phosphate (Ca_4_[PO_4_]_2_O [TTCP], Matexcel, Shirley, NY, USA, CAT#CER-0016) and calcium hydrogen phosphate (CaHPO_4_, Sigma-Aldrich, CAT#1021441000), then further mixing it with a 1:1 weight of CaCO_3_ (Sigma-Aldrich, CAT#21061). This powder, mixed with 10% gelatine (Sigma-Aldrich, CAT#9000-70-8) for 5 min, was spread thinly over clingfilm and left to dry overnight at 30 °C. The dried HAA ground using a mortar and pestle was passed through a multi-layered sieve to obtain particles within the 200–300 µm range. The resultant micro-scale particles were crosslinked in 0.1% glutaraldehyde (Sigma-Aldrich, CAT#111-30-8) solution for 10 min, washed thrice with distilled water, soaked in phosphate-buffered saline (PBS, Gibco, Waltham, MA, USA, CAT#2156340), and dried by incubating at 60 °C overnight. The treated HAA particles were transferred into glass vials and autoclaved, then immersed aseptically in DMEM/F12 serum-free medium for 24 h ahead of use.

### 2.3. AlamarBlue Assay

The AlamarBlue assay (ThermoFisher, CAT#10099022) was used to assess the viability of MSCs when cultured with HAA. The MSCs were cultured in a 96-well plate without or with 20–30 HAA particles, and the medium was changed every 3 days. The effect of HAA on MSC viability was assessed every 24 h for 144 h by replacing the culture medium with 10% AlamarBlue (made in the full serum medium), incubating cultures for a further 4 h and reading the relative fluorescence units (RFU) using excitation (ex)560 nm and emission (em)590 nm in a FLUOstar Omega microplate reader (BMG LABTECH, Ortenberg, Germany) [20].

### 2.4. CCK-8 Assay

The viability of MSCs incubated without or with HAA was also assessed every 2 days for 6 days by replacing the culture medium with 10% Cell Counting Kit (CCK)-8 reagent made in the full serum medium (CCK-8 test kit [Sigma-Aldrich, CAT#B34304]), incubating for a further 4 h, before reading the optical density (OD) at 450 nm using a FLUOstar Omega microplate reader.

### 2.5. Scanning Electron Microscopy

The scanning electron microscope (SEM) was used to analyse MSCs co-cultured without or with HAA for 10 days. The MSCs fixed with a 2.5% glutaraldehyde made in PBS for 10 min were dehydrated via immersing for 15 min sequentially once in 30% ethanol, 50% ethanol, 70% ethanol, and 90% ethanol and twice in 100%. The dehydrated samples were rinsed once in 50% hexamethyldisilane (made in PBS), washed once in 100% hexamethyldisilane for 20 min and then incubated in 100% hexamethyldisilane overnight. The dried samples were coated with chromium using the Quorum Q150T ES (Quorum Technologies, Lewes, UK) and imaged using an S-4800 scanning electron microscope (Hitachi High-Tech, Maidenhead, UK) [5].

### 2.6. Transfection of HEK-293T Cells

HEK-293T cells were transiently transfected with either pVAX1-BMP-2, pVAX1-BMP-7 or pVAX1-BMP-2/7 expression plasmid (Addgene, Watertown, MA, USA) using jetOPTIMUS^®^ reagent (1 µL reagent per 1 µg plasmid DNA) (VWR, Lutterworth, UK), as per manufacturer’s instructions (PolyPlus Transfection, Illkirch-Graffenstaden, France) [20]. At least 24 h after the transfection, the secretory BMP-2, BMP-7 or BMP-2/7 in the full serum spent medium was assessed by using Human BMP-2 (R&D Systems, Minneapolis, MN, USA, CAT#DY355) and Human BMP-7 (R&D Systems, USA, CAT#DY354) DuoSet ELISA kits.

### 2.7. Preparation of a HAA + MSC + HEK Bone Graft for Implantation

#### 2.7.1. Co-Culturing of HEK-293T/MSCs on HAA

A total of 10–15 mg of HAA particles (200–300 µM in size) per well were added to wells of a 96-well plate, then 2 × 10^5^ cells of a 1:1 ratio of MSCs and HEK-293Ts expressing BMP-2/7 in 100 µL culture medium were added to each well. The culture medium was replaced with the full serum medium every 3 days, and the formation of scaffold nodules was observed by microscopy. After 1 week, the scaffold nodules were harvested into microfuge tubes. Also, on days 1, 7, and 14, cells cultured with HAA scaffold were stained with 50 μg/mL Hoechst 33342 (Invitrogen, Carlsbad, CA, USA, CAT#H1399) and 4 μM Calcein AM (Invitrogen, CAT#2387265) and then imaged the fluorescence staining by confocal microscopy.

#### 2.7.2. Decellularization of HAA Scaffolds Co-Cultured with MSC-HEK Cells

The harvested HAA scaffold nodules were decellularized by subjecting them to 3 cycles of freezing at −80 °C for 4 h and thawing at room temperature [21,22]. The decellularized scaffolds were then dried using the Scanvac Coolsafe 110 freeze dryer (LaboGene A/S, Allerød, Denmark).

#### 2.7.3. Estimating BMP-2/7 Levels in Decellularized HAA Scaffolds

The decellularized HAA scaffolds were hydrated in 500 µL PBS/well and the concentrations of BMP-2 and BMP-7 in the cultured medium were quantified using Human BMP-2 DuoSet ELISA and Human BMP-7 DuoSet ELISA, respectively, according to the manufacturer’s instructions. The OD at 450 nm was recorded using the FLUOstar Omega microplate reader.

#### 2.7.4. Fabrication of Implanting Material

The harvested decellularized HAA particles were processed using a freeze-drying method and subsequently packed into perforated plastic moulds. The material was then compressed into cuboidal blocks measuring 2 × 2 × 2 mm^3^, with an estimated density of 13.3 mg/mm^3^ [23].

#### 2.7.5. In Vivo Model Preparation

The in vivo tests were performed, as described, at Huazhong University (Wuhan, China) (Research Ethics Approval; IACUC#3294). A total of 36 mice (C57BL/6J) aged 9 weeks (Shulaibao Biotechnology Co., Ltd., Wuhan, China) were randomly divided into three groups (Table 1). After grouping, ear punching was performed for identification and recording purposes. The mice were housed in the animal experimental facility, and the animal cages were cleaned every 3 days. The mice were provided with food and water on a scheduled basis, and their activity was observed.

#### 2.7.6. Implantation

The mice were anaesthetized with 0.3% sodium barbiturate (Sigma-Aldrich, CAT#4390-16-3). The fur on both hind limbs was carefully shaved and disinfected with 0.2% Iodophor Sanitizer (Jiangxi Caoshanhu Disinfection Supplies Co. Ltd., Nanchang, China). The muscle gap in the anterior tibial region was dissected until the tibia was adequately exposed, facilitating the implantation of the HAA scaffolding block into each leg. The muscle and skin were carefully sutured and disinfected with 0.2% Iodophor Sanitizer. After anaesthetic recovery, the mice were returned to labelled cages for ongoing care and maintenance at Huazhong Science and Technical University Animal Centre (Wuhan, China).

#### 2.7.7. Sample Harvesting

At each specific time point (days 7, 14, and 28), a cohort of 4 mice was selected for each experimental group (HAA, HAA + MSC, and HAA + MSC + HEK). Following humane euthanasia via cervical dislocation, the left tibia and surrounding muscle were harvested, then fixed and stored in 4% formaldehyde in PBS for further analysis. The right tibias were used for RNA extraction and real-time quantitative polymerase chain reaction (RT-qPCR) analysis.

#### 2.7.8. Micro-CT Analysis

Following fixation, each left tibia was wrapped in parafilm and scanned using a SkyScan 1176 X-ray microtomography system (Bruker MicroCT, Kontich, Belgium) at a voltage of 58 kV and a current of 431 µA. The scanning process employed an image pixel size of 9 and a rotation of 0.3, with a high-resolution exposure time of 1000 ms [5]. The appendages were unwrapped and returned to a 10% formalin solution and stored at a temperature of 4 °C. The resultant samples were prepared for histological staining and analysis. The obtained CT data were used for 3D modelling and quantitative analysis of new bone formation.

#### 2.7.9. RT-qPCR Analysis

The right tibias harvested from the mice were processed immediately upon collection. The gastrocnemius muscles and fibula were removed, and the tibia, along with the surrounding tissue around the scaffold, was trimmed, then 1 g of tissue fragments was rapidly frozen using liquid nitrogen and crushed by mortar and pestle. The crushed tissue was homogenized using the VCX150 sonicator (Sonics & Materials Inc., Newtown, CT, USA).

The extraction of RNA from the homogenized tissue was performed using the High-Capacity cDNA Reverse Transcription Kit (Applied Biosystems, Waltham, MA, USA, CAT#4374966), as per the manufacturer’s protocol. Briefly, 2 μg of isolated RNA was converted to cDNA by incubating the reaction mixture in a Thermal cycler (T100TM BIO-RAD, Watford, UK), for 10 min at 42 °C, followed by 10 min at 25 °C, 120 min at 37 °C and 5 min at 85 °C. The concentration and purification of the cDNA were assessed using a Nanodrop One spectrophotometer (Thermo Fisher Scientific, Loughborough, UK). The cDNA was stored at−20 °C for later use [25].

The expression levels of BMP-2 and BMP-7 in the cDNA were estimated using qPCR. The forward and reverse primers (100 µM) were diluted at a ratio of 1:20 with nuclease-free water (Merck, Beijing, China, CAT#7732-18-5) (Table 2). The cDNA samples were also diluted at a ratio of 1:20 with nuclease-free water. The master mix was prepared by combining 1 μL of the diluted cDNA sample with 1 μL of nuclease-free water, and 5 μL of 2X SYBR Green (SYBR™ Green PCR Master Mix, Thermo Fisher Scientific, Loughborough, UK, CAT#4309155) per well for future usage. The forward and reverse diluted primers were subsequently mixed at a 1:1 ratio. In each well of the plate, 8 μL of the master mix and 2 μL of the primer mixture were loaded at 4 °C. The reaction was run using the CFX Connect™ PCR (BIO-RAD, UK) as outlined in Table 3, and the plate was configured for real-time reaction monitoring. The relative change in the target gene expression was calculated using the 2^−ΔΔCq^ method, where ΔCq is the difference between the quantification cycle (Cq) of the target gene and the Cq of the reference gene GAPDH and ΔΔCq is the difference between ΔCq of untreated and ΔCq of treated [25].

#### 2.7.10. Histology of Undecalcified Samples

The left limb from each group at a particular time point (days 7, 14, and 28) was embedded in polymethyl methacrylate (PMMA) following serial dehydration. The embedded samples were cut into 10 μm sections using the EXAKT 300 CP microtome (Band System, Norderstedt, Germany), and undecalcified sections were subjected to Masson–Goldner’s trichrome staining [26]. These processes were outsourced from AICA Sci-tech Ltd. (Beijing, China). The histological sections were then imaged using a light microscope (Zeiss; Carl Zeiss Microscopy GmbH, Jena, Germany). The overall gross appearance of the specimens was imaged using a TM-DM10 imaging system (TOMLOV, Los Angeles, CA, USA).

### 2.8. Statistical Analysis

Statistical analyses were performed using IBM SPSS Statistics software v.28.0 (IBM Corp., Armonk, NY, USA) and GraphPad Prism 9.5.0 (GraphPad Software Inc., San Diego, CA, USA). All data are presented as mean ± standard deviation (SD). T-tests were used when comparing two groups in parametric, and a 2-way analysis of variance (ANOVA) was performed with Tukey’s post hoc test. A *p*-value < 0.05 was considered statistically significant.

## 3. Results

### 3.1. Scaffold Production and Biocompatibility

**Figure 1:**
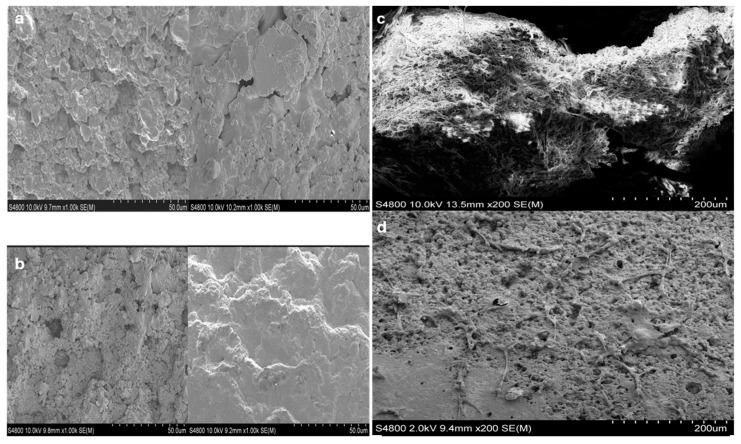
Visualizing the HAA scaffold surface structure using SEM [24]. HAA scaffold samples were fixed with 4% glutaraldehyde and dehydrated ahead of SEM analysis. Images denote the surface structure of the HAA scaffold at ×200 (**a**) and ×1000 (**b**) magnification. (**c**) The MSCs were cultured on the HAA scaffold for 10 days, as illustrated by the fibrous structures stretched across the surface. (**d**) A small number of cells adhered to the surface of HAA initially. The length of the scale bar represents 50 µm (**a**,**b**) or 200 µm (**c**,**d**). Reprinted from Ref. [24].

When MSCs seeded in 96-well plates were approximately 80% confluent, they were co-cultured with HAA particles for 10 days. The cells migrated from the 96-well to adhere to the scaffold particles, were then fixed, dehydrated, dried, and subjected to SEM imaging. The surface of the HAA particles appeared to be rough and irregular under microscopic examination (Figure 1a,b). There were visible pores and crevices distributed throughout the surface. We observed numerous MSCs migrating from the 96-well plates and adhering and aggregating on the surface of these scaffold particles, with cell extensions penetrating the interstices of the scaffold surface (Figure 1c). The three-dimensional (3D) structure of the HAA particles vividly highlights the aggregation of MSCs onto the scaffold and their migration to the non-contact surface of the particles, demonstrating the migratory capacity of MSCs and showcasing their excellent interfacial affinity for HAA. Figure 1d shows that the surface of the HAA scaffold particles became densely covered with cells after co-culturing them with the cells for approximately two weeks.

The HAA is made of HA, TTCP, and CaHPO_4_ [15]. Following printing, the structure retains its integrity through gelatine crosslinking, which forms a stabilizing matrix that holds the powdered components together prior to the hydroxyapatite-forming reaction. Biologically, gelatine enhances the scaffold’s compatibility with tissue by mimicking collagen, the main organic component of bone, and improving its capacity to bind osteoinductive proteins such as BMPs. In addition, the formation of HA through the reaction between TTCP and CaHPO_4_ provides the scaffold with strong osteoconductive properties, promoting cell adhesion, proliferation, and new bone formation.

**Figure 2:**
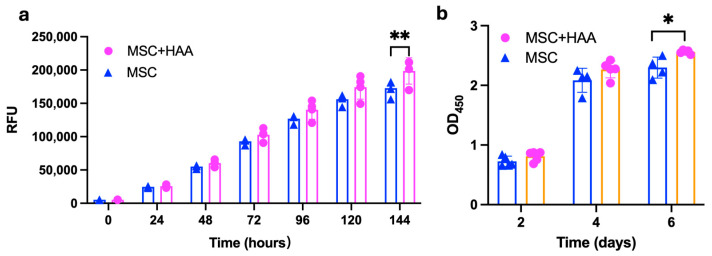
The effect of HAA scaffold on MSCs viability. The viability of MSCs co-cultured with HAA scaffold was assessed via the AlamarBlue assay (**a**) and CCK-8 test (**b**). Each bar represents the mean value of the test performed in quadruplicates, and error bars indicate standard deviation (SD). A two-way ANOVA was conducted to determine the statistical significance of the overall effect. The MSC + HAA group exhibited a significantly higher proliferation rate compared to the MSC-only group on day 6 in the CCK-8 assay (*p* < 0.01). Consistently, the MSC + HAA group showed significantly greater proliferation than the MSC group on day 7 in the Alamar Blue assay (*p* < 0.05). (*, *p* < 0.05; **, *p* < 0.01). Reprinted from Ref. [24].

The AlamarBlue and CCK8 assays were employed to assess the biocompatibility of HAA with MSCs. The results indicated no difference (Figure 2). According to the AlamarBlue assay, no statistically significant difference was observed between the proliferation of MSCs and MSCs with HAA, suggesting that the presence of the HAA particles was not cytotoxic (Figure 2a). Similarly, the CCK8 test indicated no difference in MSC viability in the absence or presence of HAA (Figure 2b). These assays have suggested that HAA possesses favourable biocompatibility with MSCs, thus confirming that the material is non-cytotoxic and, therefore, can be used to support cell growth in vitro.

### 3.2. Expression of BMP-2/7 in HEK-293T Cells

**Figure 3:**
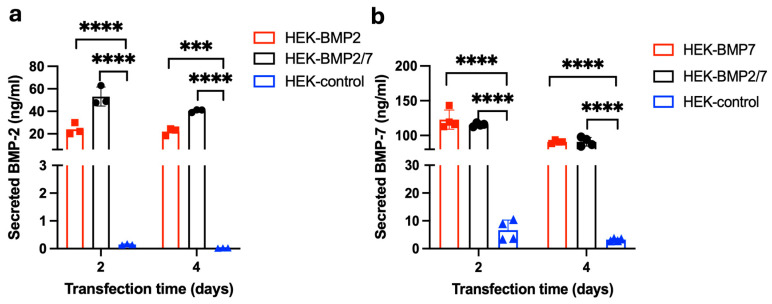
Analysis of BMP-2 and BMP-7 secretion from HEK-293T cells. HEK-293T cells were transfected with the plasmid encoding BMP-2, BMP-7 or BMP-2/7 and analysed the secretion of BMP-2 and BMP-7 after 48 h and 96 h of transfection by using BMP-2 or BMP-7 ELISA. (**a**) The concentrations of secreted BMP-2 in HEK expressing BMP-2 or BMP2/7 and the HEK-control (without plasmid) group. (**b**) The concentrations of secreted BMP-7 in HEK expressing BMP-7 or BMP2/7 and the HEK-control (without plasmid) group. BMP-2/7 group showed significantly higher BMP-2 and BMP-7 secretion compared to the control, BMP-2 group had significantly higher BMP-2 secretion than the control (*p* < 0.0001), BMP-7 group also had significantly higher BMP-2 secretion than the control (*p* < 0.0001). (***, *p* < 0.001; ****, *p* < 0.0001). Reprinted from Ref. [24].

HEK-293T cells were selected for expressing BMP-2/7 as these cells were shown to express BMP-2/BMP-7 in the past [27,28]. HEK-293T transfected with the BMP-2 expression plasmid, but not an empty vector, secreted 26.038 ± 5.594 ng/mL at 48 h and 23.89 ± 0.722 ng/mL at 96 h of BMP-2 (Figure 3a). HEK-293T transfected with the BMP-2/7 expression plasmid also secreted 48.242 ± 0.793 ng/mL at 48 h and 40.001 ± 1.654 ng/mL at 96 h of BMP-2 (Figure 3a). Figure 3b depicts HEK-293T cells transfected with the BMP-7 expression plasmid secreted 131.038 ± 16.906 ng/mL at 48 h and 92.121 ± 2.238 ng/mL at 96 h of BMP-7 in the medium. HEK-293T cells transfected with the BMP-2/7 expression plasmid also secreted 116.626 ± 2.279 ng/mL in the medium at 48 h and 96.111 ± 2.368 ng/mL at 96 h of BMP-7 (Figure 3b).

### 3.3. Characterization of the HAA + MSC + HEK Bone Graft by Confocal Microscopy

**Figure 4:**
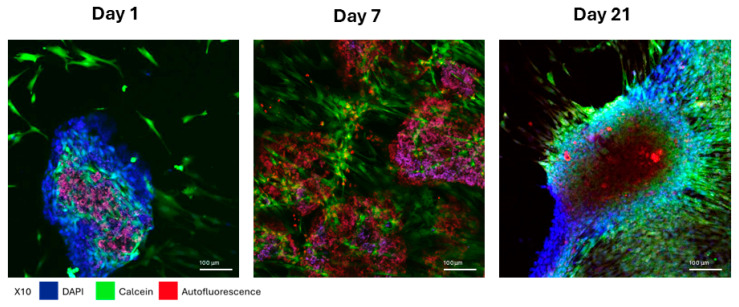
Morphological changes in MSCs adhering to HAA scaffold in full serum medium. The cell morphology of MSCs and HEK 293T cells co-cultured on HAA scaffolds in a normal growth medium for 1, 7, or 21 days was imaged by confocal fluorescence microscopy. The blue for DAPI fluorescence, green for Calcein fluorescence, and red for the scaffold autofluorescence. The length of the scale bar represents 100 µm. Reprinted from Ref. [24].

Cells cultured on the HAA scaffold are shown in Figure 4. On the first day following attachment of MSCs and HEK 293-T cells (expressing BMP-2/7) to the HAA scaffold surface was incomplete. By day 7, increased cell proliferation resulted in more extensive cell attachment to the scaffolds. By day 14, nearly the entire scaffold was enveloped by cells in all samples. Calcein AM fluorescence staining of cells is shown in green, whereas the scaffold autofluorescence is shown in red.

### 3.4. The Surface Morphology and BMP-2/7 Levels in the Decellularized HAA Scaffold

**Figure 5:**
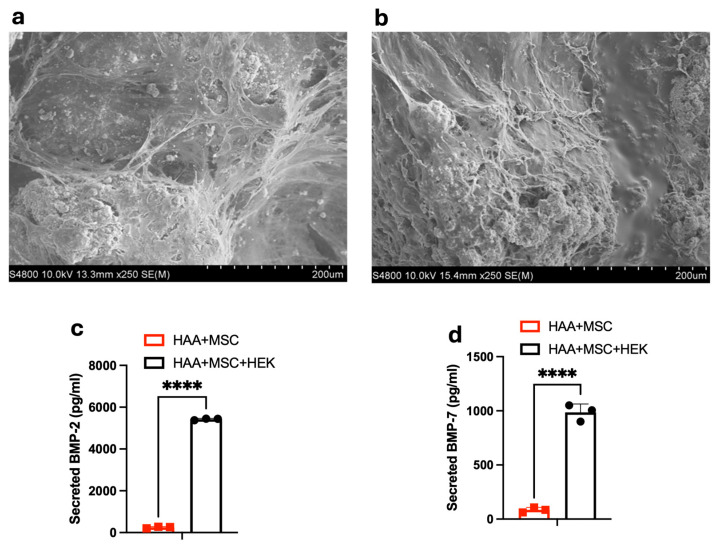
The effects of HAA + MSC + HEK decellularization on the scaffold morphology and BMP-2/7 levels. MSCs and HEK-293T cells co-cultured with the HAA scaffold for 14 days underwent visualization by the SEM before (**a**) and after (**b**) decellularization. ELISA was used to assess the secreted BMP-2 (**c**) and BMP-7 (**d**) levels in the spent cell culture media of HAA + MSC and HAA + MSC + HEK cultured for 3 days before decellularization. The length of the scale bar represents 200 µm. The HAA + MSC + HEK group showed significantly higher BMP-2 and BMP-7 levels compared to the HAA + MSC group (*p* < 0.0001). (****, *p* < 0.0001). Reprinted from Ref. [24].

SEM was employed to elucidate the microstructural details of the osteogenic scaffold surfaces. The images effectively illustrate the surfaces of scaffolds bearing adherent cells which formed multiple layers, highlighting discernible morphological differences. At a magnification of 2000× (Figure 5a,b), distinctive fibre-shaped and spherical structures were observed. In the decellularized samples, the collagen fibres exhibited a slender appearance compared to their previous aspect, and the spherical structures were no longer apparent. After decellularization, the normal cellular structures disappeared, leaving behind some residual decellularized fragment-like tissue. The ELISA outcomes for the decellularized osteogenic scaffolds indicate a significant presence of both BMP-2 and BMP-7 (Figure 5c,d).

### 3.5. Analysis of Micro-CT of the Implanted HAA + MSC + HEK Bone Graft

**Figure 6:**
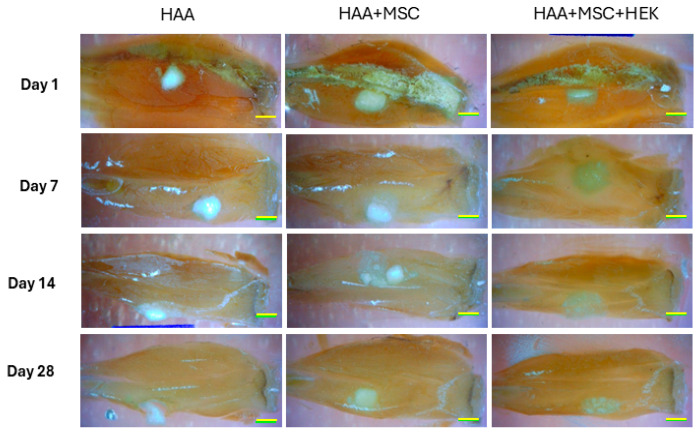
Overall observation of new bone formation in mice models. The observation on the whole of new bone formation following implanting of HAA, HAA + MSC, and HAA + MSC + HEK at days 1, 7, 14, and 28. The length of the scale bar represents 2 mm. Reprinted from Ref. [24].

The gross appearance of sections of the implants (HAA scaffold control, MSCs cultured onto HAA [HAA + MSC] or MSCs and HEK293-T cells expressing BMB2/7 cultured onto HAA [HAA + MSC + HEK]) in tissue is shown in Figure 6. The surface views reveal the presence of the scaffold material, which remains visible, along with callus formation at various time points, specifically on days 1, 7, 14, and 28. Notably, the group implanted with the HAA + MSC + HEK bone graft exhibited a more pronounced yellowish stain, indicating the presence of the osteogenic scaffold. This was characterized by richer tissue colouration due to a greater influx of migrating cells.

**Figure 7:**
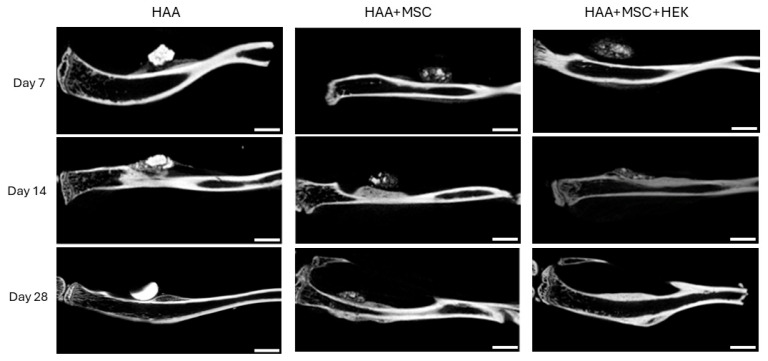
Micro-CT scans of bone and calluses of mice post-operation. The new bone formation following implanting of HAA, HAA + MSC, and HAA + MSC + HEK at days 1, 7, 14, and 28 was analysed by micro-CT. In the control group, the implants were absorbed slowly and showed stronger density. The implanted HA/CC exhibited greater density and lacked the pores resulting from decellularization, hindering osteocyte infiltration and reducing the absorbability and degradation of the implanted material. The length of the scale bar represents 2 mm. Reprinted from Ref. [24].

Micro-CT imaging demonstrated varying degrees of callus formation around osteogenic scaffolds of different compositions in different groups within the tibiae of mice (Figure 7). The quantity of bone formed and the quality and density of newly formed callus varied across different time points. Overall, there was a progressive increase in new bone formation across all groups. Compared to the HAA group, the HAA + MSC and HAA + MSC + HEK groups displayed richer and denser peri-nodular osteogenesis, validated through subsequent software-based quantitative analyses. Additionally, it was observed that in the HAA group, osteogenic scaffolds exhibited higher density, shrinking in size over time with minimal changes in density. Conversely, the HAA + MSC and HAA + MSC + HEK groups showed lower osteogenic scaffold density, implying cellular and soft tissue ingrowth. This suggests that osteogenic scaffolds subjected to cell adhesion and decellularization processes attracted cell adhesion and ingrowth, with the decrease in density indicating a faster degradation rate compared to the unmodified HAA scaffold.

**Figure 8:**
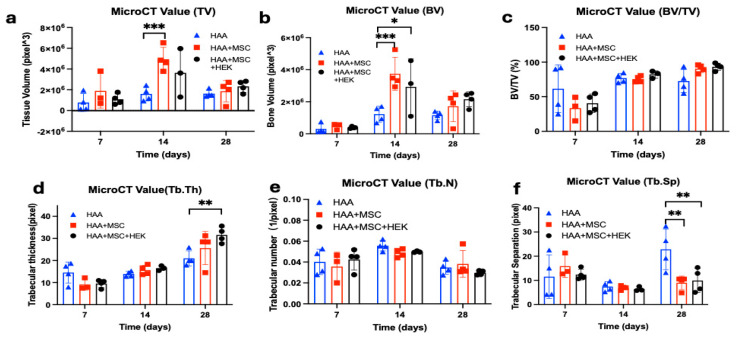
Micro-CT analysis of the quality of bone formation in mice implanted with HAA-based bone grafts. Various qualities (**a**–**f**) of bone formation were assessed by micro-CT amongst the test groups of mice consisting of the control HAA, MSC + HAA, and HEK + MSC + HAA groups on days 7, 14, and 28 post-implantation. The qualities of bone formation explored are tissue volume (TV (**a**)), bone volume (BV (**b**)), bone volume/tissue volume (BV/TV (**c**)), trabecular thickness [Tb.Th (**d**)), trabecular number (Tb.N (**e**)), and trabecular separation (Tb.Sp (**f**)). Statistically significant differences are marked as *, *p* < 0.05; **, *p* < 0.01, ***, *p* <0.001. Reprinted from Ref. [24].

Various qualities of bone formation in mice implanted with HAA-based bone grafts were assessed by micro-CT (Figure 8). The tissue (Figure 8a) and bone volumes (Figure 8b) were shown to peak on day 14, with a significant difference between the HAA and HAA + MSC groups (*p* = 0.0005). The value for bone volume subsequently decreases by day 28; on the other hand, statistically significant differences were observed between the control HAA scaffold and HAA + MSC (*p* = 0.0002) and HAA + MSC + HEK (*p* = 0.0166). The percentage of bone volume in tissue volume (BV/TV) showed no significant differences between groups across the time points (Figure 8c). However, the overall trend indicates a consistent increase in BV/TV percentage across all groups over time.

In regard to trabecular thickness (Tb.Th), no statistically significant difference (*p* > 0.05) was observed among the groups on either day 7 or day 14 of the study (Figure 8d). However, a notable trend emerged, showing an increase in Tb. Th measurements across the three time points. Day 28 was the sole time point at which significant differences materialized (*p* = 0.0015). Further analysis utilizing Tukey’s multiple comparisons tests revealed significant disparities, specifically between the HAA and HAA + MSC + HEK groups (*p* = 0.0015).

The analysis also showed changes in trabecular number (Tb. N, Figure 8e) and trabecular separation (Tb.Sp, Figure 8f), wherein the Tb.Sp at day 28 exhibited contrasting results to bone volume (Figure 8b). Specifically, the HAA group exhibited a significant difference compared to the HAA + MSC + HEK group (*p* = 0.0034) and HAA + MSC group (*p* = 0.0017). This disparity in Tb.Sp, which was inversely related to osteogenesis, reflects larger gaps between trabeculae, indicative of lower-quality callus formation. These findings suggest favourable osteogenic outcomes in the HAA + MSC + HEK group.

Based on the application of analytical software, HAA particles can be readily identified and distinguished in early-stage specimens using micro-CT. HAA particles appear as discrete high-density granules, whereas osteogenesis is characterized by a continuous, lower-density band-like region of newly formed bone. By integrating the results of Figure 8b,d, it can be concluded that the HAA + MSC + HEK group generated more bone tissue and internal trabecular structures, indicating that this group exhibited superior overall osteogenic capacity.

### 3.6. Histological Observations

**Figure 9:**
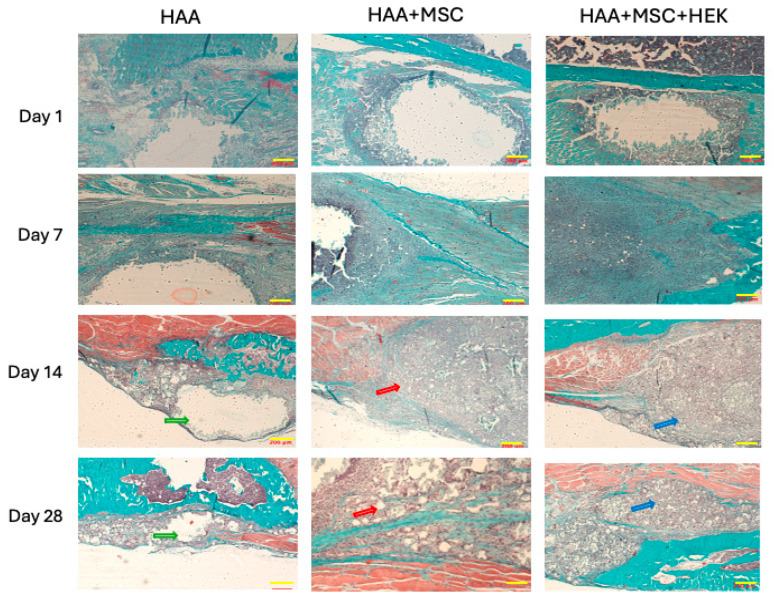
Analysing time-dependent osteogenesis by Masson–Goldner’s trichrome-staining. Masson-Goldner’s trichrome staining of tibial scaffold grafts in three groups mentioned above on day 1, 7, 14, and 28 following the implantation. Muscle fibres are stained in red, cell nuclei in a grey-blue hue, and fibres and newly formed bone are highlighted in green. Scale bars (200 µm) are shown in yellow in each image. The length of the scale bar represents 200 µm. Reprinted from Ref. [24].

Masson–Goldner’s trichrome staining was used to assess the progress of osteogenesis in the various experimental groups at different time points (Figure 9). Little difference was observed among the HAA, HAA + MSC, and HAA + MSC + HEK groups on days 1 and 7. Notably, at these time points, voids could be seen at the scaffold implantation sites due to the scaffold washing out during section staining, accompanied by mild tissue reactions and cell aggregations, demonstrating similar patterns across the groups. However, by days 14 and 28, all three groups showed cellular ingress at the scaffold sites, displaying fibrous proliferation and new bone formation indicated by green staining. Increased deposition of collagen fibres (indicated by green staining) by day 28 suggests a progression in cellular differentiation or increased osteoblastic activity, despite the emergence of smaller voids. Inter-group differences were notably manifested, with increased green staining and reduced voids in the HAA + MSC and HAA + MSC + HEK groups compared to the control, indicating less cellular ingress at the implantation sites in the control group compared to the other two groups. as indicated by green arrows, corresponding to scaffold degradation and resorption within the host tissue. In contrast, no prominent voids were observed in the HAA + MSC and HAA + MSC + HEK groups at the same time points, as indicated by the red and blue arrows, respectively. This indicates that implants in these groups were more readily absorbed and that the scaffold area was increasingly occupied by host or transplanted cells. Moreover, in the day 28 sections, the regions indicated by blue arrows exhibited higher cellular density and more intense purple staining compared to those marked by red arrows, indicating a greater extent of osteochondral tissue formation and enhanced osteogenesis in the HAA + MSC + HEK group.

Toluidine Blue staining of tibial scaffold grafts revealed comparable osteogenic conditions among the HAA, HAA + MSC, and HAA + MSC + HEK groups on days 1 and 7 (Figure S4). Similarly, unstained voids appeared at the implantation sites due to the detachment of scaffold material during staining. We observed increased cellular ingress, fibrous proliferation and heightened osteogenic activity in these areas, implying a greater potential for subsequent new bone formation. Inter-group comparisons indicated that both the HAA + MSC and HAA + MSC + HEK groups exhibited increased cellular ingress and deeper bluish-purple staining in the later stages compared to the HAA group. Groups implanted with cell-processed material demonstrate enhanced cell attraction and phagocytic absorption. Moreover, the increased presence of stimulated cells secreting fibrous tissue suggested potential osteoblastic differentiation.

### 3.7. Expression of Osteogenic Markers: BMP-2 and BMP-7

**Figure 10:**
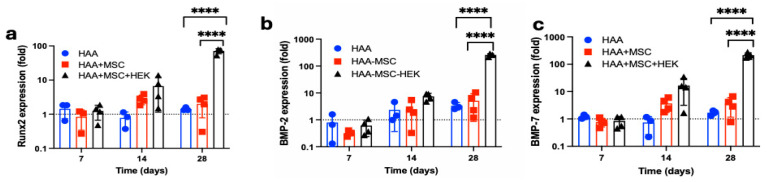
Analysis of the expression of the osteogenic markers: BMP-2 and BMP-7 in the implanted tissues. Total RNA was isolated from the implanted region in the right tibia on days 7, 14, and 28 of the post-implantations. The gene expression of Runx2 (**a**), BMP-2 (**b**), and BMP-7 (**c**) were analysed via RT-qPCR. The expression values are normalized to the expression of the housekeeping gene GAPDH. ****, *p* < 0.0001. Reprinted from Ref. [24].

The gene expression of osteogenic biomarkers Runx2 (Figure 10a), BMP-2 (Figure 10b) and BMP-7 (Figure 10c) was analysed by RT-qPCR at days 7, 14, and 28 after implantation HAA, HAA + MSC, and HAA + MSC + HEK (Figure 10). All three biomarkers, Runx2, BMP-2, and BMP-7, exhibited consistent increases in expression, with the HAA + MSC + HEK group displaying the highest expression of Runx2, BMP-2, and BMP-7 on day 28 of the study. All three osteogenic biomarkers–RunX2, BMP-2, and BMP-7–showed highest expression on day 28, correlating with the peak BV/TV (Figure 8c) and Tb.Th (Figure 8d) observed in the HAA + MSC + HEK group acaot the time point.

## 4. Discussion

In this study, we successfully prepared BMP-2/7-secreting HEK-293T cells after exogenously expressing BMP-2/7 in them, which were co-cultured with human umbilical cord matrix MSCs on HAA to form an in vitro engineered construct. After decellularization, this construct was assessed both in vitro and in vivo to evaluate the osteogenesis capacity in comparison with the HAA + MSC group and HAA control group. MSCs were transduced with BMP-2/7 genes using the lentivirus method (Figure S1), but the resulting BMP2 or BMP7 expression in the transduced MSCs was lower than anticipated (Figure S2). As a result, HEK-293T cells were selected as an alternative expression platform. Micro-CT results revealed that the overall trend indicated an increase in new bone formation in all three groups as time progressed. In comparison to the HAA group, the HAA + MSC and HAA + MSC + HEK groups exhibited a more abundant and denser bone formation surrounding the implants (Figure 7), and these findings were further validated in subsequent osteogenic gene expression and histology analysis. In the micro-CT analysis, the HAA group served as a reference for evaluating the osteogenic capacity of a scaffold material lacking both cells and active osteogenic factors. In similar experiments conducted by our group, the sham group presented in the Emma’s study more clearly demonstrated that the absence of scaffold material leads to impaired bone defect healing [19]. The micro-CT results further indicated that the presence of cell-active components in the experimental groups contributed to enhanced bone regeneration outcomes.

Human allograft has long been recognized as the most suitable alternative for autologous bone graft substitutes. Allografts not only contain inorganic and organic bone matrix but also growth factors, especially BMPs [29]. BMPs in bone tissue are not in their free form but are stored in bone matrix and osteoid. They are released during bone turnover, where the bone matrix is resorbed by osteoclasts; the BMPs are then released to act upon stem cells to induce bone regeneration [29,30]. To use free-form recombinant human BMPs clinically, very high doses are required [31]. To mitigate the need for such high doses, integrating BMPs into the bone matrix to mimic an allograft is preferable. In this study, we aimed to enhance MSCs to express BMPs and incorporate them into their extracellular matrix. Adenoviruses, retroviruses, lentiviruses and adeno-associated viruses (AAVs) are commonly used as viral transduction of human MSCs [32]. They are highly efficient, but there are concerns about the safety of integrating viral vectors and the potential immunogenicity of viral antigens. [33] Nonviral delivery methods may offer a safer alternative to viral delivery, but they are often associated with extremely low transfection efficiency and high toxicity, which make them a less favourable technique for engineering MSC therapies [33,34,35].

Our findings have shown that human MSCs are particularly difficult to transfect with plasmids encoding the genes for BMP-2/7. This is consistent with the existing literature where it has been reported that rAAV gene vector transfection of human bone marrow stromal cells (hBMSCs), with transfection efficiencies reportedly ranging from 8% to 52% [34,35]. This is lower than the transfection efficiency observed in other cell types using viral transfection methods [36]. To address this issue, HEK-293T cells were selected as they are highly amenable to various transfection methods including chemical, physical, and viral approaches. Their robust nature allows for an efficient uptake and expression of exogenous DNA, RNA, or other molecules that could achieve more than 95% transfection [37,38].

Throughout our experimental process, we noticed remarkably high efficiency of BMP-2/7 gene transfection using HEK cells; ELISA analyses revealed that both BMP-2 and BMP-7 were released into the culture medium. Thus, we hypothesized that by establishing a co-culture of HEK-293T and MSCs, the BMP-2/7 expressed would be able to integrate into the extracellular matrix. Consistent findings suggest that allografts are successful bone graft substitutes [39,40]. Allografts could provide both organic and inorganic bone matrix for host bone regeneration after implantation. The drawback of using allograft clinically is their limited resource, as well as the potential of disease transmission and allergic responses [41]. However, in comparison to transplant-engineered synthetic biomaterials with living stem cells which require strict regulatory control, it will be more practical to mimic an allograft where no living cells are required. HEK-293T are an immortalised cell line derived from human embryonic kidney tissue. HEK-293T is a sublineage of the original HEK293 cell line, transfected with a plasmid carrying the SV40 T-antigen origin of replication that can lead to large copy numbers of mRNA transcripts to produce large quantities of recombinant proteins. The studies addressed the safety concerns associated with HEK cells by employing decellularization or more advanced methods to remove immunogenic antigens from HEK cells. This innovative approach requires further experiments to validate its effectiveness, and determine potential genetic safety, and necessitates longer-term experimental observations for confirmation and potential improvement.

Several vaccines employed in Europe and the United States during the COVID-19 pandemic were developed using genetically modified organisms (GMOs) derived from HEK cells, suggesting that HEK cell-based GMOs may hold promise for broader clinical applications in the future [42]. Notably, the present animal study did not reveal any significant interspecies immunological responses. Moreover, countries such as Japan and members of the European Union have already established legal frameworks permitting the clinical use of various GMO-based therapies [43].

This can be produced through the engineering of osteogenic stem cells on biodegradable biomaterials, followed by decellularization to leave behind only the extracellular matrix [15]. These biomaterials should be biodegradable and have a composition similar to that found in natural bone [44]. In this context, HAA is a very suitable biomaterial to produce artificial allografts [5]. By combining human umbilical cord matrix MSCs and HEK BMP-2/7 transfected cells with HAA, an in vitro bone engineering construct was produced. Following decellularization, the construct was tested non-toxic, containing BMP-2/7 at a similar level as allograft, and promoted bone regeneration in the in vivo tests.

The decellularization process of allograft is well-established [45,46]. This method is also extended to engineered bone, ligament, and tendon [47,48]. We applied a simple decellularization process for this study with satisfactory results; however, further optimization and refinement of methodologies may be employed for future studies and potential clinical applications [49,50]. The decellularized allograft products were implanted on the surface of the tibia of murine models without bone defects, this study aimed to demonstrate enhanced osteogenesis rather than the effect of bone defect repair [51,52,53]. Evidence suggests that there is a special type of osteogenic stem cells in the periosteum which can form new bone rapidly [54,55]. Our results showed that osteogenesis was significantly enhanced in the HAA + MSC + HEK group than in the HAA group when they were implanted on the surface of the tibia. However, further studies using bone defect models and large animals may be required.

The transfection methods require further refinement and optimization, ideally by directly transfecting MSCs through improved plasmid design, exploring more efficient promoters, and selecting cell lines with enhanced BMP expression levels [56]. Secondly, reliance on small animal models like rodents poses challenges in directly translating results to human scenarios. Employing moderately larger models in future studies could aid the improvement of simulation accuracy. Finally, while scaffold granules offer enhanced cell adhesion, they lack the spatial structures found in 3D-printed scaffolds [57,58]. Research indicates that spatial structure influences bone cell behaviour, underscoring the need for 3D-printed scaffolds with controlled structures [59,60]. These advancements would better align with clinical trial requirements and enhance translational potential.

In this study, we utilized a complex structure incorporating transfected HEK293T cells to facilitate the sustained release of osteoinductive factors such as BMP-2/7. While the initial gene delivery efficiency may be lower compared to the direct application of mesenchymal stromal cells (MSCs), the use of HEK293T cells offers several distinct advantages. These include high reproducibility, human-like post-translational modifications, and the ability to function as a controllable delivery system for therapeutic proteins. Additionally, this approach allows for the generation of decellularized scaffolds containing bioactive extracellular matrix components, thereby eliminating the need for living cells and reducing associated immunogenic and regulatory concerns.

Although MSCs are widely recognized for their regenerative capacity, they are subject to donor variability, differentiation uncertainty, and viability constraints. By contrast, the HEK293T-based strategy, despite its relative complexity, enables a more standardized and potentially better method for bioactive scaffold generation. Further optimization of transfection efficiency and structural design is expected to enhance performance in future applications.

## 5. Conclusions

This study demonstrated that the integration of MSC and HEK cells expressing BMP-2/7 with HA/CC scaffolds enhances osteogenesis, indicated by increased bone formation and elevated osteogenic gene expression. Micro-CT and histological analyses confirmed that BMP-2/7 expressing HEK cells significantly improved bone density and distribution. The scaffold’s bioactive properties, combined with BMP-2 and BMP-7, enabled a reduced BMP dosage while maintaining efficacy. Future studies should focus on evaluating the scaffold performance in load-bearing or critical-size defect models, larger animal models, and 3D-printed scaffolds to better simulate human applications for bone defect treatment. Future studies should also focus on comparing HAA with other calcium phosphate-based materials, such as β-TCP, which would be useful in determining whether similar osteogenic responses can be achieved across different material compositions.

## Figures and Tables

**Table 1:** Experimental mouse groups. Reprinted from Ref. [24].

Groups	Control (HAA)	HAA + MSC (Decellularized MSC Matrix)	HAA + MSC + HEK (Decellularized MSC + HEK BMP2/7 Matrix)
Day 7	4	4	4
Day 14	4	4	4
Day 28	4	4	4

**Table 2:** RT-PCR Forward and Reverse Primers. Reprinted from Ref. [24].

Primer Name	Sequence
BMP-2	FORWARD: 5′-TGTATCGCAGGCACTCAGGTCA-3′
	REVERSE: 5′CGGGTTGTTTTCCCACTCGT-3′
BMP-7	FORWARD: 5′-TTCGTCAACCTCGTGGAACA-3′
	REVERSE: 5′-ACGTCTCATTGTCGAAGCGT-3′
GAPDH	FORWARD: 5′-CTCATGACCACAGTCGATGCC-3′
	REVERSE: 5′-GGGATGACCTTGCCCACAG-3′

**Table 3:** qPCR Running Conditions. Reprinted from Ref. [24].

Step		Temperature	Time
Initial denaturation		95 °C	5 min
Denaturation	×39	95 °C	15 s
Annealing		60 °C	15 s
Extension		72 °C	1 min
Melt curve		95 °C	15 s
		58 °C	1 min
		95 °C	15 s

## Data Availability

The original contributions presented in the study are included in the article/Supplementary Material, further inquiries can be directed to the corresponding author.

## Supplementary Materials

The following supporting information can be downloaded at https://www.mdpi.com/article/10.3390/jfb16100361/s1; Figure S1: Analysing the expression of BMP-2 and BMP-7 by Confocal microscopy and flow cytometry in MSCs through lentiviral transduction. MSCs were transduced with lentiviral particles carrying BMP-2/GFP or BMP-7/mCherry, which are co-expressed by using the internal ribosome entry site (IRES) and a single promoter (EF1α). After 48 h, the fluorescent protein expression was visualised by Confocal microscopy (**a**,**c**) and quantified by flow cytometry (**b**,**d**). (**a**,**c**), images of GFP (corresponding to BMP-2) and mCherry (corresponding to BMP-7), respectively, the magnification is 20×. (**b**,**d**), The flow cytometry histogram showing the percentage of MSCs that were successfully transfected by GFP (BMP-2) and mCherry (BMP-7), respectively. The plots represent three independent experiments. Figure S2: Analysing the expression of BMP-2/7 by ELISA in MSCs transfected with lentivirus carrying BMP-2 or BMP-7. The lysates of MSCs transduced for 48 hours without (MSCs) or with BMP-2 (MSCs-BMP2 under CMV promoter or MSCs-BMP2 under EF1α promoter) or BMP-7 (MSCs-BMP7 [CMV]/MSCs-BMP7 [EF1α]) lentiviral particles were used in ELISA to assess BMP-2 (**a**) or BMP-7 (**b**) expression. Figure S3: Analysing the secretion of BMP-2/7 in MSCs transduced with lentivirus carrying BMP-2 or BMP-7 by ELISA. The cultured media of MSCs transduced for 48 hours without (MSCs) or with BMP-2 (MSCs-BMP2 [CMV]) or BMP-7 (MSCs-BMP7 [CMV]) lentiviral particles were used in ELISA to assess BMP-2 (**a**) or BMP-7 (**b**) secretion. Figure S4: Toluidine Blue staining of the implanted tibial scaffolds. Toluidine Blue staining of osteogenesis at the tibial scaffold implantation sites in mice across different experimental groups and time points. Chondrocytes and osteogenic cells appear bluish-purple against a pale blue background. Scale bars (200 µm) are shown in yellow.

## References

[1] Betz Randal R. (2002). Limitations of Autograft and Allograft: New Synthetic Solutions. Orthopedics.

[2] Lomas R., Chandrasekar A., Board T.N. (2013). Bone allograft in the U.K.: Perceptions and realities. HIP Int..

[3] Baldwin P., Li D.J., Auston D.A., Mir H.S., Yoon R.S., Koval K.J. (2019). Autograft, Allograft, and Bone Graft Substitutes: Clinical Evidence and Indications for Use in the Setting of Orthopaedic Trauma Surgery. J. Orthop. Trauma..

[4] Buser Z., Brodke D.S., Youssef J.A., Meisel H.J., Myhre S.L., Hashimoto R., Park J.B., Tim Yoon S., Wang J.C. (2016). Synthetic bone graft versus autograft or allograft for spinal fusion: A systematic review. J. Neurosurg. Spine.

[5] Shi Y., He R., Deng X., Shao Z., Deganello D., Yan C., Xia Z. (2020). Three-dimensional biofabrication of an aragonite-enriched self-hardening bone graft substitute and assessment of its osteogenicity in vitro and in vivo. Biomater. Transl..

[6] Ielo I., Calabrese G., De Luca G., Conoci S. (2022). Recent Advances in Hydroxyapatite-Based Biocomposites for Bone Tissue Regeneration in Orthopedics. Int. J. Mol. Sci..

[7] Gao C., Peng S., Feng P., Shuai C. (2017). Bone biomaterials and interactions with stem cells. Bone Res..

[8] Kobayashi M., Nihonmatsu S., Okawara T., Onuki H., Sakagami H., Nakajima H., Takeishi H., Shimada J. (2019). Adhesion and Proliferation of Osteoblastic Cells on Hydroxyapatite-dispersed Ti-based Composite Plate. In Vivo.

[9] Zhong Q., Li W., Su X., Li G., Zhou Y., Kundu S.C., Yao J., Cai Y. (2016). Degradation pattern of porous CaCO_3_ and hydroxyapatite microspheres in vitro and in vivo for potential application in bone tissue engineering. Colloids Surf. B Biointerfaces.

[10] Suarez C.E., McElwain T.F. (2010). Transfection systems for Babesia bovis: A review of methods for the transient and stable expression of exogenous genes. Vet. Parasitol..

[11] Chen D., Ji X., Harris M.A., Feng J.Q., Karsenty G., Celeste A.J., Rosen V., Mundy G.R., Harris S.E. (1998). Differential Roles for Bone Morphogenetic Protein (BMP) Receptor Type IB and IA in Differentiation and Specification of Mesenchymal Precursor Cells to Osteoblast and Adipocyte Lineages. J. Cell Biol..

[12] Tzameli I., Fang H., Ollero M., Shi H., Hamm J.K., Kievit P., Hollenberg A.N., Flier J.S. (2004). Regulated Production of a Peroxisome Proliferator-activated Receptor-γ Ligand during an Early Phase of Adipocyte Differentiation in 3T3-L1 Adipocytes*. J. Biol. Chem..

[13] Nishimura R., Hata K., Matsubara T., Wakabayashi M., Yoneda T. (2012). Regulation of bone and cartilage development by network between BMP signalling and transcription factors. J. Biochem..

[14] McDonald N.M., Woodell-May J.E., Pietrzak W.S. Bone morphogenetic protein concentration in human demineralized bone matrix. Proceedings of the 51st Annual Meeting of the Orthopaedic Research Society.

[15] Steijvers E., Ghei A., Xia Z. (2022). Manufacturing artificial bone allografts: A perspective. Biomater. Transl..

[16] Cahill K.S., McCormick P.C., Levi A.D. (2015). A comprehensive assessment of the risk of bone morphogenetic protein use in spinal fusion surgery and postoperative cancer diagnosis. J. Neurosurg. Spine.

[17] Wang Q., Zhang Y., Li B., Chen L. (2017). Controlled dual delivery of low doses of BMP-2 and VEGF in a silk fibroin–nanohydroxyapatite scaffold for vascularized bone regeneration. J. Mater. Chem. B.

[18] Bez M., Pelled G., Gazit D. (2020). BMP gene delivery for skeletal tissue regeneration. Bone.

[19] Steijvers E., Shi Y., Lu H., Zhang W., Zhang Y., Zhao F., Wang B., Hughes L., Barralet J.E., Degli-Alessandrini G. (2025). Rapid assessment of the osteogenic capacity of hydroxyapatite/aragonite using a murine tibial periosteal ossification model. Bioact. Mater..

[20] Jannoo R., Xia Z., Row P.E., Kanamarlapudi V. (2023). Targeting of the Interleukin-13 Receptor (IL-13R)alpha2 Expressing Prostate Cancer by a Novel Hybrid Lytic Peptide. Biomolecules.

[21] Nonaka P.N., Campillo N., Uriarte J.J., Garreta E., Melo E., de Oliveira L.V., Navajas D., Farre R. (2014). Effects of freezing/thawing on the mechanical properties of decellularized lungs. J. Biomed. Mater. Res. A.

[22] Xing Q., Yates K., Tahtinen M., Shearier E., Qian Z., Zhao F. (2015). Decellularization of fibroblast cell sheets for natural extracellular matrix scaffold preparation. Tissue Eng. Part. C Methods.

[23] Liu B., Zhou X. (2021). Freeze-Drying of Proteins. Methods Mol. Biol..

[24] Lu H. (2024). Enhanced Osteogenesis by Combining Exogenous BMPs with Hydroxyapatite/Calcium Carbonate Bone Grafts: In Vitro and In Vivo Study. M.D. Thesis.

[25] Kanamarlapudi V., Tamaddon-Jahromi S., Murphy K. (2022). ADP-ribosylation factor 6 expression increase in oesophageal adenocarcinoma suggests a potential biomarker role for it. PLoS ONE.

[26] Gruber H.E., Ingram J.A., An Y.H., Martin K.L. (2003). Basic Staining and Histochemical Techniques and Immunohistochemical Localizations Using Bone Sections. Handbook of Histology Methods for Bone and Cartilage.

[27] Chitty D.W., Tremblay R.G., Ribecco-Lutkiewicz M., Haukenfrers J., Zurakowski B., Massie B., Sikorska M., Bani-Yaghoub M. (2012). Development of BMP7-producing human cells, using a third generation lentiviral gene delivery system. J. Neurosci. Methods.

[28] Bustos-Valenzuela J.C., Halcsik E., Bassi E.J., Demasi M.A., Granjeiro J.M., Sogayar M.C. (2010). Expression, purification, bioactivity, and partial characterization of a recombinant human bone morphogenetic protein-7 produced in human 293T cells. Mol. Biotechnol..

[29] Blokhuis T.J., Lindner T. (2008). Allograft and bone morphogenetic proteins: An overview. Injury.

[30] Regauer M., Jurgens I., Kotsianos D., Stutzle H., Mutschler W., Schieker M. (2005). New-bone formation by osteogenic protein-1 and autogenic bone marrow in a critical tibial defect model in sheep. Zentralbl. Chir..

[31] Okubo Y., Bessho K., Fujimura K., Iizuka T., Miyatake S.I. (2001). In vitro and in vivo studies of a bone morphogenetic protein-2 expressing adenoviral vector. J. Bone Jt. Surg. Am..

[32] Christoffers S., Seiler L., Wiebe E., Blume C. (2024). Possibilities and efficiency of MSC co-transfection for gene therapy. Stem Cell Res. Ther..

[33] Hamann A., Nguyen A., Pannier A.K. (2019). Nucleic acid delivery to mesenchymal stem cells: A review of nonviral methods and applications. J. Biol. Eng..

[34] Zhang H., Tang X., Wang C., Sun L. (2018). AB0063 High-efficiency transduction of mesenchymal stem cells by aav2/dj vector for their potential use in autoimmune diseases. Ann. Rheum. Dis..

[35] Stender S., Murphy M., O’Brien T., Stengaard C., Ulrich-Vinther M., Søballe K., Barry F. (2007). Adeno-associated viral vector transduction of human mesenchymal stem cells. Eur. Cell Mater..

[36] Gill K.P., Denham M. (2020). Optimized Transgene Delivery Using Third-Generation Lentiviruses. Curr. Protoc. Mol. Biol..

[37] Tan E., Chin C.S.H., Lim Z.F.S., Ng S.K. (2021). HEK293 Cell Line as a Platform to Produce Recombinant Proteins and Viral Vectors. Front. Bioeng. Biotechnol..

[38] Pulix M., Lukashchuk V., Smith D.C., Dickson A.J. (2021). Molecular characterization of HEK293 cells as emerging versatile cell factories. Curr. Opin. Biotechnol..

[39] Cheng I., Oshtory R., Wildstein M.S. (2007). The Role of Osteobiologics in Spinal Deformity. Neurosurg. Clin. North. Am..

[40] Viola A., Appiah J., Donnally C.J., Kim Y.H., Shenoy K. (2022). Bone Graft Options in Spinal Fusion: A Review of Current Options and the Use of Mesenchymal Cellular Bone Matrices. World Neurosurg..

[41] Carlisle E.R., Fischgrund J.S., Errico T.J., Lonner B.S., Moulton A.W. (2009). CHAPTER 27—Bone Graft and Fusion Enhancement. Surgical Management of Spinal Deformities.

[42] Lao T., Avalos I., Rodríguez E.M., Zamora Y., Rodriguez A., Ramón A., Alvarez Y., Cabrales A., Andújar I., González L.J. (2023). Production and characterization of a chimeric antigen, based on nucleocapsid of SARS-CoV-2 fused to the extracellular domain of human CD154 in HEK-293 cells as a vaccine candidate against COVID-19. PLoS ONE.

[43] Kauffmann F., Van Damme P., Leroux-Roels G., Vandermeulen C., Berthels N., Beuneu C., Mali S. (2019). Clinical trials with GMO-containing vaccines in Europe: Status and regulatory framework. Vaccine.

[44] Von Euw S., Wang Y., Laurent G., Drouet C., Babonneau F., Nassif N., Azaïs T. (2019). Bone mineral: New insights into its chemical composition. Sci. Rep..

[45] Amini Z., Lari R. (2021). A systematic review of decellularized allograft and xenograft-derived scaffolds in bone tissue regeneration. Tissue Cell.

[46] Cowell K., Statham P., Sagoo G.S., Chandler J.H., Herbert A., Rooney P., Wilcox R.K., Fermor H.L. (2023). Cost-effectiveness of decellularised bone allograft compared with fresh-frozen bone allograft for acetabular impaction bone grafting during a revision hip arthroplasty in the UK. BMJ Open.

[47] Blaudez F., Ivanovski S., Hamlet S., Vaquette C. (2020). An overview of decellularisation techniques of native tissues and tissue engineered products for bone, ligament and tendon regeneration. Methods.

[48] Fisher J.N., Peretti G.M., Scotti C. (2016). Stem Cells for Bone Regeneration: From Cell-Based Therapies to Decellularised Engineered Extracellular Matrices. Stem Cells Int..

[49] Tamayo-Angorrilla M., López de Andrés J., Jiménez G., Marchal J.A. (2022). The biomimetic extracellular matrix: A therapeutic tool for breast cancer research. Transl. Res..

[50] León-Félix C.M., Maranhão A.Q., Amorim C.A., Lucci C.M. (2024). Optimizing Decellularization of Bovine Ovarian Tissue: Toward a Transplantable Artificial Ovary Scaffold with Minimized Residual Toxicity and Preserved Extracellular Matrix Morphology. Cells Tissues Organs.

[51] Marsell R., Einhorn T.A. (2011). The biology of fracture healing. Injury.

[52] Berendsen A.D., Olsen B.R. (2015). Bone development. Bone.

[53] Batoon L., Millard S.M., Wullschleger M.E., Preda C., Wu A.C., Kaur S., Tseng H.W., Hume D.A., Levesque J.P., Raggatt L.J. (2019). CD169(+) macrophages are critical for osteoblast maintenance and promote intramembranous and endochondral ossification during bone repair. Biomaterials.

[54] Duchamp de Lageneste O., Julien A., Abou-Khalil R., Frangi G., Carvalho C., Cagnard N., Cordier C., Conway S.J., Colnot C. (2018). Periosteum contains skeletal stem cells with high bone regenerative potential controlled by Periostin. Nat. Commun..

[55] Roberts S.J., van Gastel N., Carmeliet G., Luyten F.P. (2015). Uncovering the periosteum for skeletal regeneration: The stem cell that lies beneath. Bone.

[56] Chen W., Zhang C., Wu Y., Su X. (2019). Soluble expression and purification of high-bioactivity recombinant human bone morphogenetic protein-2 by codon optimisation in *Escherichia coli*. Protein Eng. Des. Sel..

[57] Wang Y., Xie C., Zhang Z., Liu H., Xu H., Peng Z., Liu C., Li J., Wang C., Xu T. (2022). 3D Printed Integrated Bionic Oxygenated Scaffold for Bone Regeneration. ACS Appl. Mater. Interfaces.

[58] Ben Shabat Y., Fischer A. (2015). Design of Porous Micro-Structures Using Curvature Analysis for Additive-Manufacturing. Procedia CIRP.

[59] Zhang X.-Y., Yan X.-C., Fang G., Liu M. (2020). Biomechanical influence of structural variation strategies on functionally graded scaffolds constructed with triply periodic minimal surface. Addit. Manuf..

[60] Jamshidinia M., Wang L., Tong W., Kovacevic R. (2014). The bio-compatible dental implant designed by using non-stochastic porosity produced by Electron Beam Melting^®^ (EBM). J. Mater. Process. Technol..

