# Supplementary figures and images for: Enhanced Osteogenesis by Combining Exogenous BMPs with Hydroxyapatite/Aragonite Bone Grafts: In Vitro and In Vivo Studies
Source: J Funct Biomater. 2025 Sep 26;16(10):361. doi: 10.3390/jfb16100361 (PMC12565441; doi:10.3390/jfb16100361)

## Slide 1
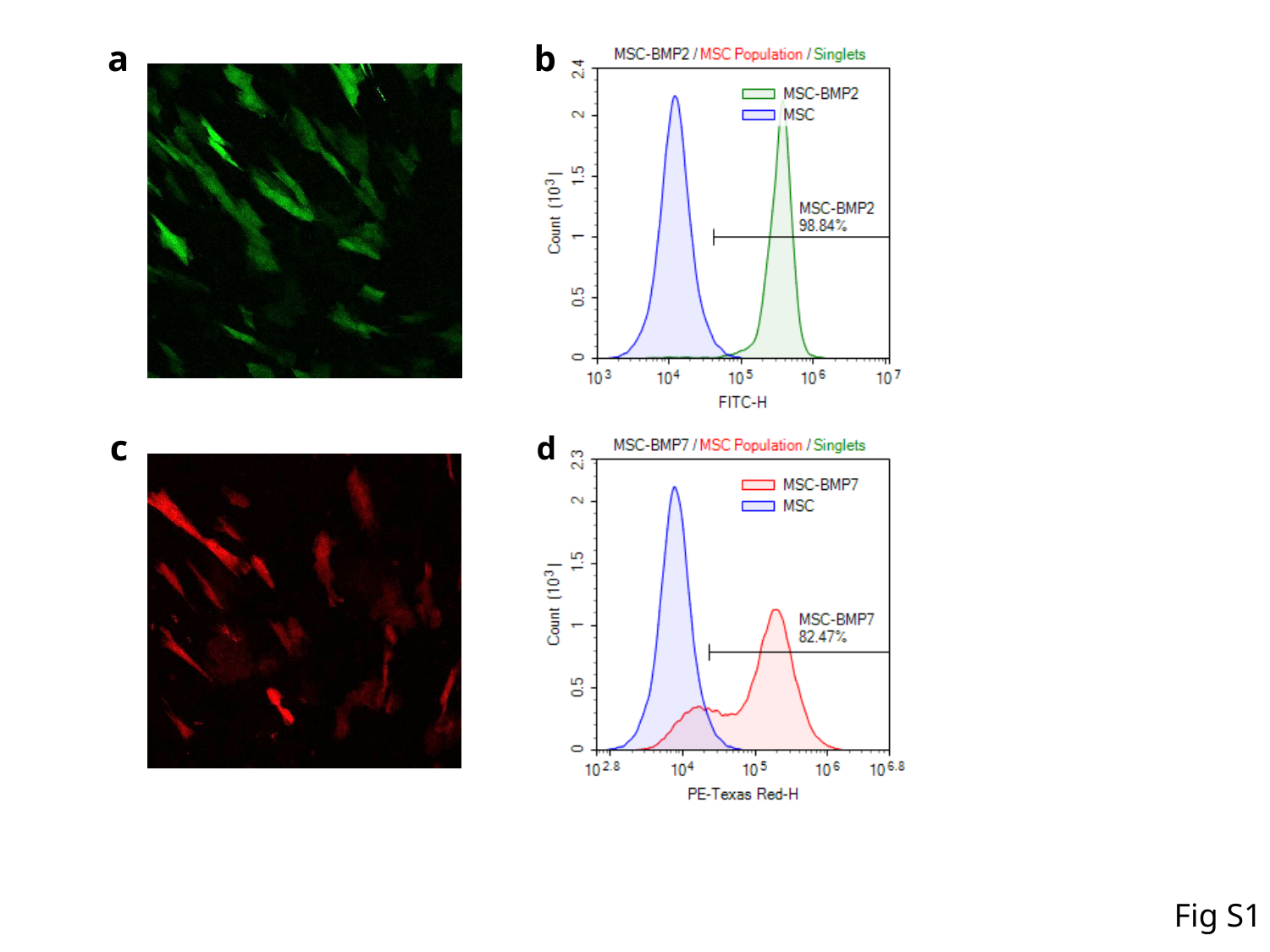

a
b
c
d
Fig S1

## Slide 2
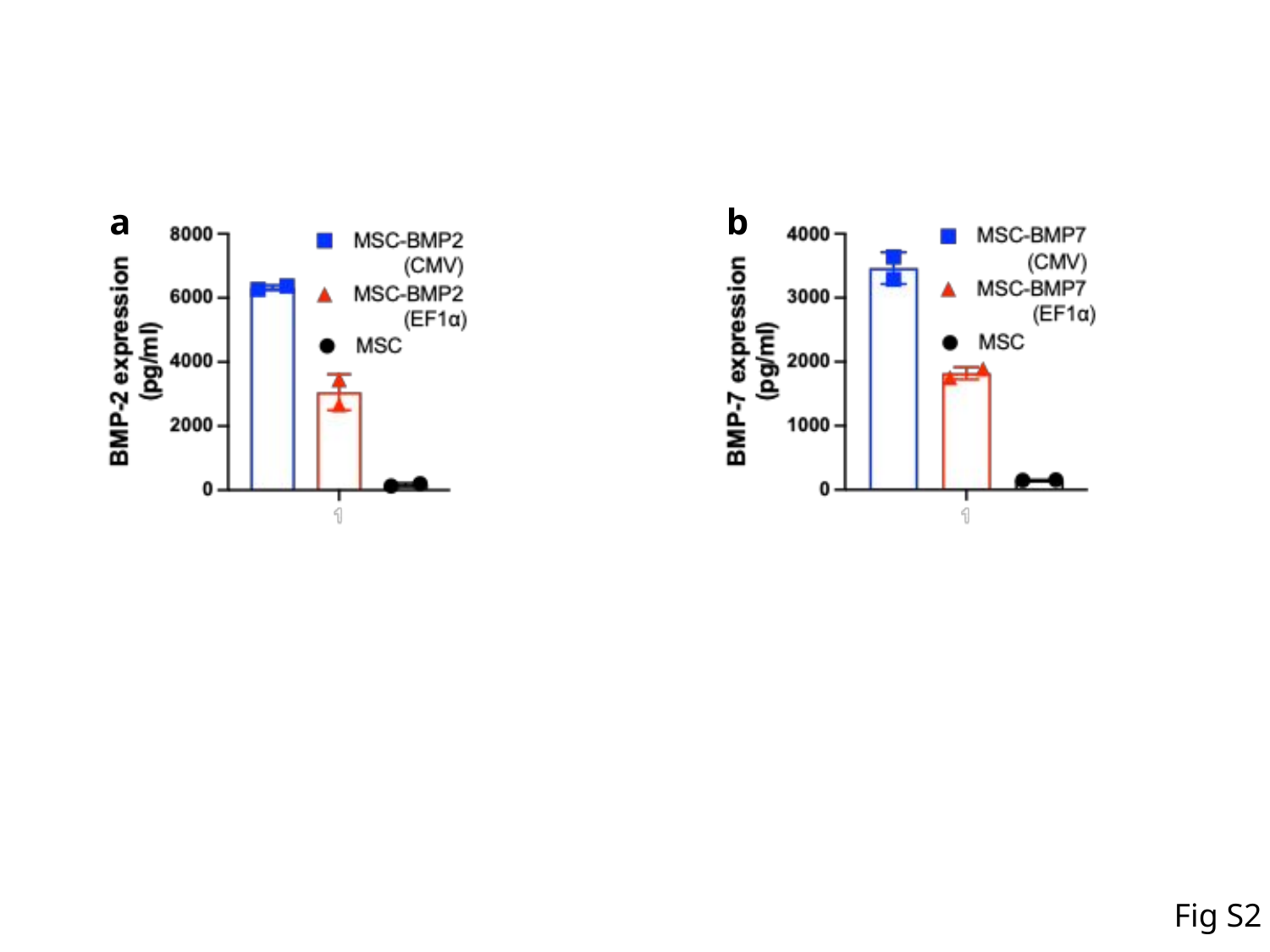

a
b
Fig S2

## Slide 3
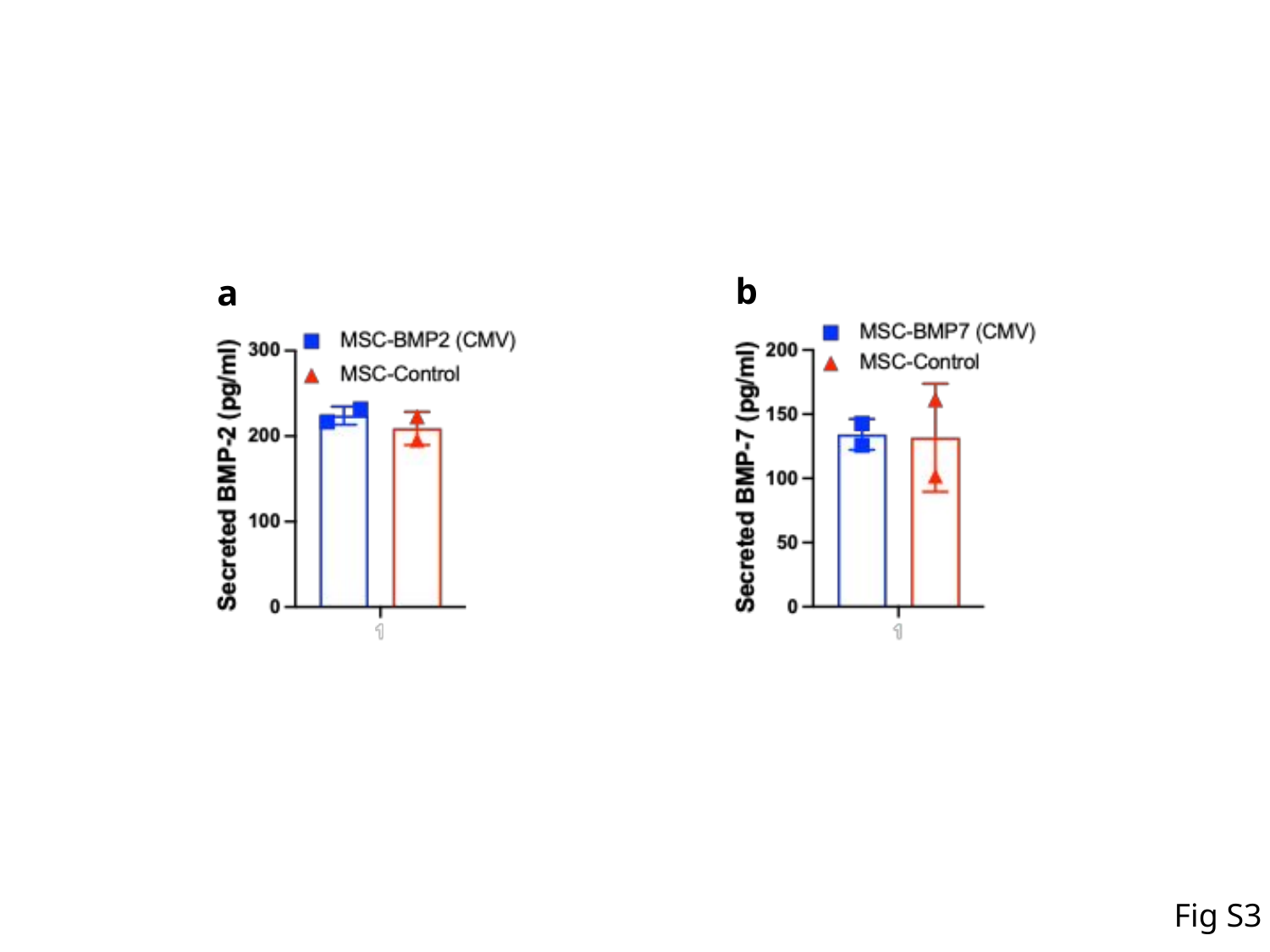

b
a
Fig S3

## Slide 4
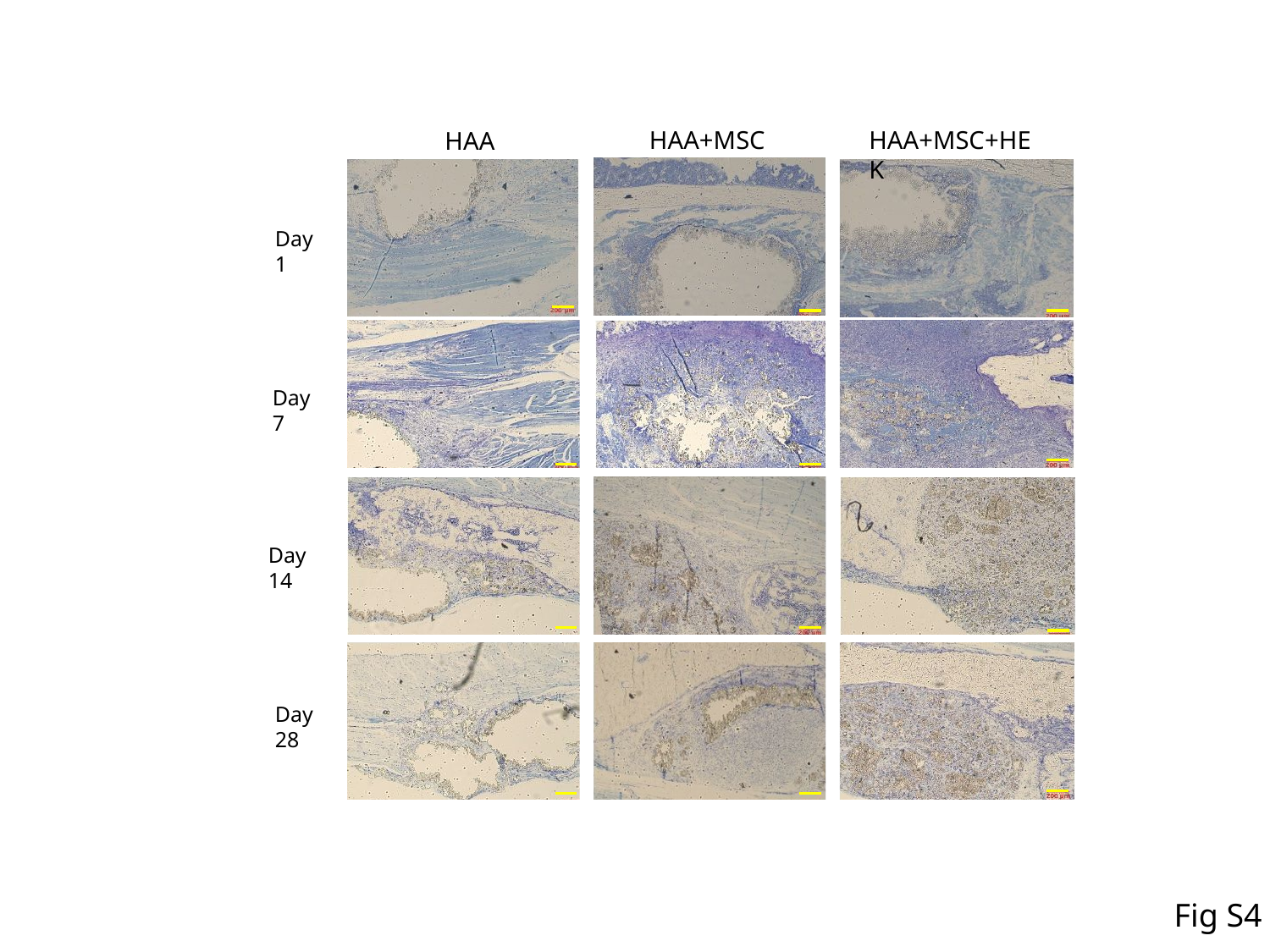

HAA+MSC
HAA+MSC+HEK
HAA
Day 1
Day 7
Day 14
Day 28
Fig S4

Supplement: Supplementary file 1 [file jfb-16-00361-s001.zip › jfb-3464694-supplementary.pptx]
